# Hybrid Self-Assembling Nanoparticles Encapsulating Zoledronic Acid: A Strategy for Fostering Their Clinical Use

**DOI:** 10.3390/ijms23095138

**Published:** 2022-05-05

**Authors:** Marianna Abate, Lorena Scotti, Valeria Nele, Michele Caraglia, Marco Biondi, Giuseppe De Rosa, Carlo Leonetti, Virginia Campani, Silvia Zappavigna, Manuela Porru

**Affiliations:** 1 Department of Precision Medicine, University of Campania “Luigi Vanvitelli”, Via Santa Maria di Costantinopoli, 16, 80138 Naples, Italy; marianna.abate@unicampania.it (M.A.); michele.caraglia@unicampania.it (M.C.); silvia.zappavigna@unicampania.it (S.Z.); 2 Department of Pharmacy, University Federico II of Naples, Via Domenico Montesano, 49, 80131 Naples, Italy; lorena.scotti@unina.it (L.S.); valeria.nele@unina.it (V.N.); mabiondi@unina.it (M.B.); gderosa@unina.it (G.D.R.); 3 Research and Advanced Technologies Department, IRCCS Regina Elena National Cancer Institute, Via Elio Chianesi, 53, 00144 Rome, Italy; carlo.leonetti@ifo.it (C.L.); manuela.porru@ifo.it (M.P.)

**Keywords:** self-assembling nanoparticles, bisphosphonates, zoledronic acid, lyophilization, differential scanning calorimetry, glioblastoma, scale-up

## Abstract

Self-assembling nanoparticles (SANPs) promise an effective delivery of bisphosphonates or microRNAs in the treatment of glioblastoma (GBM) and are obtained through the sequential mixing of four components immediately before use. The self-assembling approach facilitates technology transfer, but the complexity of the SANP preparation protocol raises significant concerns in the clinical setting due to the high risk of human errors during the procedure. In this work, it was hypothesized that the SANP preparation protocol could be simplified by using freeze-dried formulations. An in-depth thermodynamic study was conducted on solutions of different cryoprotectants, namely sucrose, mannitol and trehalose, to test their ability to stabilize the produced SANPs. In addition, the ability of SANPs to deliver drugs after lyophilization was assessed on selected formulations encapsulating zoledronic acid in vitro in the T98G GBM cell line and in vivo in an orthotopic mouse model. Results showed that, after lyophilization optimization, freeze-dried SANPs encapsulating zoledronic acid could retain their delivery ability, showing a significant inhibition of T98G cell growth both in vitro and in vivo. Overall, these results suggest that freeze-drying may help boost the industrial development of SANPs for the delivery of drugs to the brain.

## 1. Introduction

Glioblastoma (GBM) is a highly aggressive and generally incurable brain tumor characterized by a rapid invasion of the entire brain [1]. Despite the relentless progress of treatment protocols, the prognosis of GBM is in most cases grim, with a devastatingly low median patient survival time (14.6 months from the diagnosis) [2,3] as well as a considerably high chance of recurrence [4].

Numerous attempts have been made to improve GBM therapeutic strategies, including the loading of low-molecular-weight chemotherapy drugs such as docetaxel [5], paclitaxel [6] and doxorubicin [7] within nanoparticles (NPs) for brain targeting. Among these, zoledronic acid (ZOL) is the most potent amino-bisphosphonate and showed a powerful anticancer activity in many types of tumors, including GBM [8,9,10,11]. However, the advancements in clinical outcomes have been marginal so far. This is mainly due to the unfavorable pharmacokinetic profile of ZOL that accumulates into the bone, making the achievement of tumoricidal concentration in extraskeletal tissues challenging. In addition, in the case of brain tumors, the presence of the blood–brain barrier (BBB) represents a further obstacle to hydrophilic drugs such as ZOL, thus preventing the drugs from reaching the therapeutic target [12,13]. As such, the production of nanosystems endowed with BBB-crossing ability may open novel therapeutic opportunities in the treatment of central nervous system (CNS) tumors.

A novel formulation based on hybrid self-assembling nanoparticles (SANPs) for the delivery of anionic drugs, e.g., bisphosphonates in GBM treatment, has been recently developed [14,15,16] and patented (WO/2012/042024 A1, EP2621539A1, US20140086979) by our group. The promising results obtained by using SANPs for the delivery of bisphosphonates in GBM supported the designation of ZOL as an orphan drug by the European Medicines Agency (EMA/COMP/509421/2016) and by the Food and Drug Administration (11/29/2016) for the treatment of glioma [16]. The patented product is a SANP-based formulation that is obtained by mixing lipid and inorganic components with ZOL. SANPs spontaneously form following a set-up assembling protocol and must be immediately used after the self-assembly. This strategy has been designed to overcome scale-up issues which often limit the industrial development of nanomedicines. Therefore, ZOL-encapsulating SANPs can be prepared by using components already available on the market or easy to be prepared at industrial scales. The reconstitution of parenteral antineoplastic preparations must be carried out in a hospital pharmacy specifically equipped for these activities; international guidelines for the preparation of parenteral antineoplastic drugs detail the actions needed to minimize the risk of errors during the reconstitution. This is particularly important in the case of SANPs, which self-assemble following a complex reconstitution procedure associated with a high risk of errors. In clinical settings, the use of a single powder component that can be easily reconstituted with a solvent represents a more appealing option for SANP administration.

However, freeze-drying of lipid-based colloidal dispersions is challenging and requires the use of cryoprotectants, which can protect the lipid bilayers from the damage caused by ice crystal formation during freezing [17], probably by hampering the phase transition and consequently preventing the formation of ice crystals [18]. Lyophilized formulations are generally characterized by satisfactory stability, ease of handling in terms of shipping and storage [19,20] and increased shelf life of products such as live virus vaccines [21] and biological products [22]. Moreover, lyophilized liposomes can be stored with no refrigeration, which allows a drastic reduction in storage costs.

Here, we optimized the SANP formulation protocol to obtain a single-component powder formulation to be reconstituted with a suitable solvent immediately before administration. For this purpose, a SANP dispersion was produced and tested for its response to freeze-drying. Subsequently, a thorough thermodynamic study was performed on batches containing sucrose, mannitol and trehalose to assess the effect of the different cryoprotectants on the developed formulation. Finally, in vitro and in vivo studies were performed to evaluate whether the reconstituted freeze-dried ZOL-encapsulating SANPs possessed a comparable ability to deliver ZOL in GBM cells and tissues.

## 2. Results and Discussion

**Figure 1:**
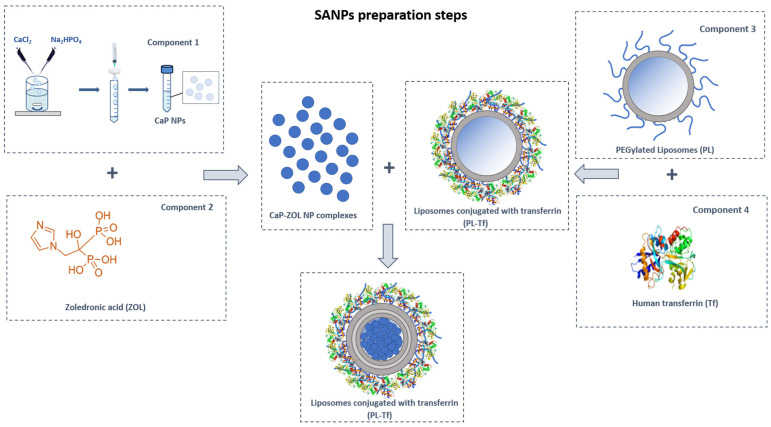
SANP preparation procedure. Preparation protocol of hybrid self-assembling NPs encapsulating ZOL (PL-Tf-CaP-ZOL/SANPs-ZOL). PL = PEGylated liposomes; Tf = transferrin; CaP NPs = calcium phosphate nanoparticles; ZOL = zoledronic acid.

The main objective of this work was the development of a strategy to simplify the preparation of ZOL-loaded SANPs to obtain SANP formulations that are easy to set up and suitable for industrial production. Specifically, the intent was to obtain a powder to be reconstituted at the time of administration with a suitable solvent. The preparation of ZOL-encapsulating SANPs usually involves three different steps (as depicted in Figure 1) and an ordered mixing of four components. Briefly, CaP NPs (component 1) are mixed with a ZOL solution to obtain CaP NP complexes. In a second step, pre-formed PEGylated cationic liposomes (component 3) are mixed with a Tf buffer solution (component 4) to obtain a colloidal dispersion of PL-Tf. SANPs are finally obtained by mixing the PL-Tf complex with CaPZ and administered straight after. In this framework, the lyophilization of SANPs in suspension was studied to select the cryoprotectant that shows the strongest propensity to preserve the stability of nanocarriers.

### 2.1. Lyophilization of SANP Formulations

In the perspective of this work, after the addition of a cryoprotectant, the mean size and size distribution of the as-produced liposomes must be retained. Indeed, lyophilization can lead to the formation of lipid aggregates [23]. Destabilizing effects such as water crystal formation during freezing, vesicle aggregation after dehydration and phase transition during rehydration do frequently occur [17]. In particular, the formation of ice in the membrane is fatal for the vesicle and must be absolutely avoided [18]. The destabilizing effect of lyophilization on liposomes in the absence of cryoprotectants is generally described in terms of increases in size and polydispersity index, which occur due to a disordered aggregation caused by the breakdown of the hydrogen bonds between the water molecules and the phospholipids [20]. Furthermore, the uncontrolled release of the encapsulated drug can occur during the secondary drying of the liposomes if the process temperature exceeds the phase transition temperature of the liposomal membrane.

A cryoprotectant can hinder membrane phase transitions, protecting the liposomes from the damage induced by ice crystallization and preventing the phase separation of the lipid components and the uncontrolled release of the loaded active molecule [18]. Cryoprotectants are generally sugars, and their protective effect relies upon two main mechanisms: (i) incorporation of sugar in place of water due to the interaction of sugar with the polar heads of the phospholipids, which is associated with a drop in the phase transition temperature of the lipid membrane; (ii) formation of a sugar matrix due to the increase in the cryoprotectant concentration in the solution during freeze-drying. This prevents vesicle melting and aggregation, as well as water crystallization upon freezing. Furthermore, the interaction between the surface of liposomes and the sugar matrix reduces the surface tension, thus stabilizing the nanocarriers after drying [24,25].

The cryoprotectant properties of sugars strongly depend on their proclivity to form a solid amorphous phase upon brisk freezing [26]. Therefore, DSC has been used to appraise the crystallization propensity of cryoprotectant solutions [27] and select the most suitable cryoprotectant among trehalose, mannitol and sucrose. In this study, DSC traces allowed the determination of thermal transitions in each sample under heating and cooling [28].

**Figure 2:**
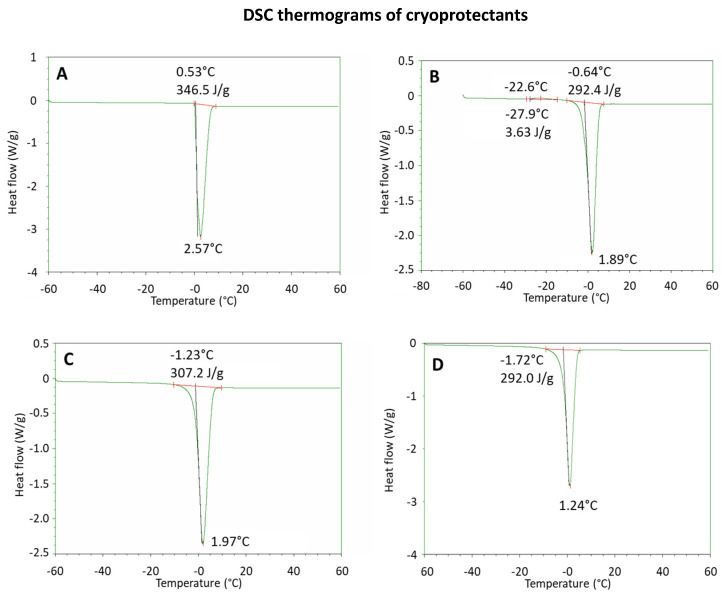
DSC studies of cryoprotectants. DSC thermograms under heating of: depurated water, used as a reference (**A**); mannitol solution (**B**); trehalose solution (**C**); sucrose solution (**D**). Exotherm is directed upward.

All DSC profiles under heating, displayed in Figure 2, exhibit an endothermic transition peak. In the case of water, used as a reference (Figure 2A and Table 1), the extrapolated average onset and peak appear at −0.57 °C and −2.58 °C, respectively, with an average heat of fusion of 338 J/g, which is consistent with literature data (334 J/g) [29].

In the presence of a cryoprotectant, the endothermic peak was broader than the water peak due to the presence of solutes (Figure 2B–D), and the extrapolated onset and peak temperature values were slightly lower compared to water, with quite lower average heats of fusion (Table 1). Differently from what has been observed with sucrose and trehalose, in the case of mannitol a small exothermic peak was identified around −20 °C, with an average heat of 3.21 J/g (Figure 2B). This peak was ascribed to a partial crystallization of mannitol during heating.

Overall, DSC results demonstrated that sucrose and trehalose possess similar thermodynamic attributes, while mannitol set out a slender propensity to crystallization, which is not ideal for an optimized lyophilized product. Therefore, mannitol was excluded from the subsequent studies.

In order to design an optimized freeze-drying process, the macroscopic collapse temperature of the formulation (T_c_) should be known [30]. T_c_ can be defined as the threshold temperature above which the freeze-dried product loses its macroscopic structure and collapses during freeze-drying [31]. T_c_ is approximately 2 °C higher than the transition glass temperature (T_g_) in the frozen state [32], or it is equal to the eutectic temperature (T_eu_) if solutes are crystallized in the frozen solution. To develop an optimized product, the operating lyophilization temperature needs to be considerably lower than T_c_ [33,34]. In this work, a “safe” process temperature was set at 10 °C below the onset temperature of the endothermic DSC peak (Table 1), i.e., at ≤−20 °C, with a “safe” pressure much lower than the vapor pressure of water at that temperature, i.e., ≤0.2 mbar.

The freeze-dried formulations were evaluated for their technological attributes, in the absence of the selected cryoprotectants (trehalose and sucrose), immediately after being rehydrated. As displayed in Table 2, a major rise in SANP size and polydispersity index (PI) was detected after lyophilization for SANPs without cryoprotectant and with trehalose.

The formulation containing sucrose showed an adequate stability in terms of mean diameter and polydispersity index. The stabilizing impact of the cryoprotectant can be associated with its binding to the liposome membrane interface which prevents membrane mobility, thereby limiting membrane disruption during freezing [35]. To avoid the formation of a cake, a rapid freezing in liquid nitrogen was necessary both to anneal the product and hamper phase separation. As displayed in Table 3, the formulation was named SANPs 1. Moreover, in order to facilitate industrial scale-up, solubilized Tf was replaced with freeze-dried Tf. Thus, an additional formulation, namely SANPs 2, was studied using solid lyophilized Tf instead of a Tf solution. In both cases, sucrose was added before lyophilization.

The SANPs 1 formulation exhibited low mean diameter and PI after lyophilization, thereby pointing at the key role of sucrose in preserving SANP stability and avoiding nanoparticle aggregation. Therefore, the lyophilized SANPs 2 (SANP 2-L) formulation was selected as the lead product for the second part of this study. To confirm the ability of the selected novel formulation to deliver ZOL, the efficacy of SANP 2-L was tested in vitro and in vivo compared to the starting SANPs 1.

### 2.2. In Vitro Effects of SANP Formulations

**Figure 3:**
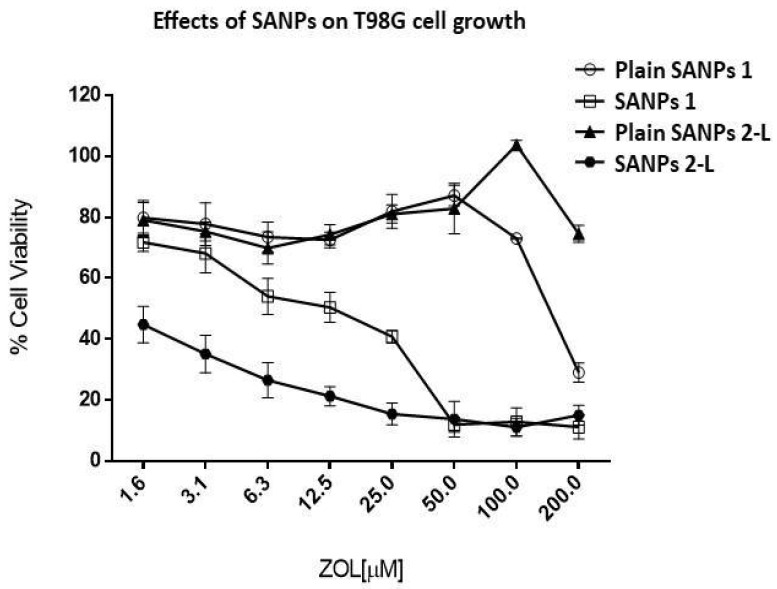
Cell viability assay on GBM cell line after treatment with the different formulations. T98G cells were seeded at a density of 2.0 × 103 cells/well in 96-well plates in serum-containing media. After 24 h incubation at 37 °C, cells were treated with increasing concentrations (1.6–200 μM) of the two different formulations SANP 1 (after preparation) and SANP 2 and the respective plain SANPs (used as reference compounds) for 72 h. MTT assay was performed as described in Section 3. The overlapping concentration points in the graph have the same percentage values of cell viability. Experiments were performed in triplicate. Data were expressed as mean value ± the standard deviation calculated for at least three repeats.

Although the SANPs showed physical–chemical characteristics appropriate for intravenous use, their cytotoxicity following the freeze-drying step was firstly investigated in vitro in a GBM cell line. In order to investigate if the lyophilized formulation maintained the ability to deliver ZOL and inhibit cell proliferation compared to the non-lyophilized one, the antiproliferative effect induced by SANPs 1 and SANP 2-L was studied on T98G cells after 72 h of incubation. Briefly, cells were seeded in 96-well plates filled with serum-containing media, and after 24 h of incubation at 37 °C, cells were treated with increasing concentrations (1.5–200 μM) of either the two different formulations or the respective plain SANPs (used as reference compounds) for 72 h. Finally, an MTT assay was performed. As reported in Figure 3, preliminary results showed that the dried formulation was characterized by a high efficacy in delivering ZOL in cells because it can significantly inhibit cell viability at very low concentrations. In more detail, plain SANPs 1 showing a percentage of cell viability lower than 80% at 200 μM but higher than 80% at 100 μM and plain SANP 2-L showing 80% cell viability at the highest concentration (200 μM) were considered nontoxic in our experimental model. Additionally, SANPs 1 inhibited 50% of cell growth (IC50) at a concentration of 12.5 µM, while freeze-dried SANP 2-L had an IC50 equal to 1.56 µM. In conclusion, we found that the freeze-drying step did not impair SANP delivery efficacy; indeed, SANP 2-L, as we supposed, enhanced ZOL delivery and consequently enhanced cell growth inhibition compared to non-freeze-dried SANPs. The highest toxicity of SANP 2-L evidenced in vitro could be reasonably ascribed to the use of freeze-dried Tf that, when mixed with the cationic liposomes, could favor the Tf/liposome interaction, leading to a higher amount of Tf conjugated on vesicle surface compared to SANPs 1. It can be assumed that the preparation procedure of SANP 2-L led to an increased interaction between nanoparticle surface and Tf, with the consequent highest amount of Tf molecules on SANPs surface and increased target activity on cell receptors. In line with these observations, a different value of zeta potential was also found for SANPs 1 and SANP 2-L (Table 3).

### 2.3. In Vivo Effects of SANP Formulations

**Figure 4:**
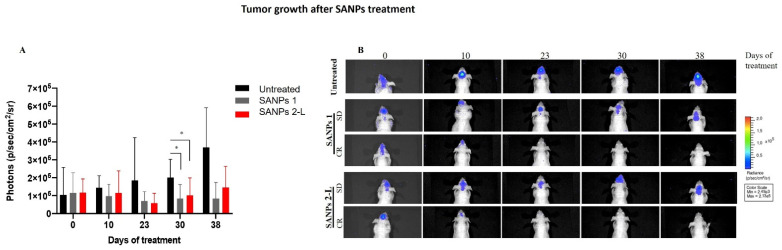
Antitumor efficacy of SANP formulation on orthotopic GBM xenograft model. U373-MG (Uppsala) LUC cells were injected into the brain of male nude mice. Real-time tumor growth was monitored using the IVIS imaging system 200 series (PerkinElmer). (**A**) Quantitative analysis of luciferase activity in vivo at various time points. Luminescent signals are expressed as mean ± SD of total flux of photons/s/cm^2^/steradian (p/s/cm^2^/sr). *p* values were calculated using an unpaired two-tailed t-test. * *p* < 0.05; *n* = 6. (**B**) Representative images of mice analyzed before administration of compounds (day 0) and during treatments on days 10, 23, 30 and 38. Mice with stable disease (SD) or complete response (CR) after SANPs 1 or SANPs 2-L treatment are shown. Data were acquired and analyzed using the Living Image software version 4.3 (PerkinElmer).

Finally, the therapeutic efficacy of the different SANP formulations was evaluated in vivo in an orthotopic GBM model. For this purpose, 2.5 × 10^5^ U373-MG (Uppsala) LUC cells were inoculated intra-brain into immunocompromised mice. This tumor model closely recapitulates a histological phenotype consistent with human GBM. After 8 days, when the tumor mass became visible by bioluminescence analysis, mice were divided into three groups: untreated mice, mice treated with SANPs 1 and with mice treated with freeze-dried SANP 2-L. Interestingly, in all cases, SANP formulations were well tolerated as no toxic deaths or body weight loss was reported in animals. The antitumor efficacy of SANP formulations was evaluated by using the IVIS imaging system. As reported in Figure 4A, tumor growth was significantly inhibited starting from 23 days after treatment with both SANP formulations that showed a very similar inhibition rate. Stabilization of the disease for about two weeks was observed in 3/6 mice, and interestingly, 1/6 mice treated with both SANPs 1 and SANP 2-L showed a complete disappearance of the tumor, as confirmed by an undetectable bioluminescent signal (Figure 4B), lasting for at least four weeks (Table 4). Compared to previous results obtained in T98G cells, in vivo studies did not show a significant difference in efficacy when using SANPs 1 and SANP 2-L. More interestingly, both the formulations maintained the ability to deliver ZOL in a brain tumor, compared to the previously developed formulation [10]. These data demonstrate the possibility to freeze-dry SANP formulations as a strategy to strengthen their clinical development, which will enable the use of ZOL for the treatment of GBM. Moreover, the possibility to replace an aqueous solution of Tf with a freeze-dried Tf further contributes to the stabilization of the formulation, making the storage of the starting components less challenging. These in vivo preliminary results confirm the efficacy of the new SANP formulation, thus supporting its potential use for the treatment of GBM.

## 3. Materials and Methods

### 3.1. Materials

Zoledronic acid (ZOL), trehalose, mannitol and sucrose were kindly provided by Lisapharma S.p.A. (Erba, Italy). 1,2-Dioleoyl-3-trimethylammonium-propane chloride (DOTAP) was kindly provided by Lipoid GmbH (Ludwigshafen, Germany); cholesterol (CHOL) and 1,2-distearoyl-sn-glycero-3-phosphoethanolamine-N-[amino(polyethylene glycol)-2000] (DSPE-PEG_2000_) were purchased from Avanti Polar (Alabaster, AL, USA). Sodium chloride, sodium hydroxide (NaOH), calcium chloride (CaCl_2_), sodium phosphate dibasic (Na_2_HPO_4_), potassium chloride and human transferrin (Tf), were purchased from Sigma-Aldrich Co. (Milan, Italy).

T98G cells were purchased from the American Type Culture Collection (ATCC). The T98G cells were cultured at 37 °C in a 5% CO_2_ atmosphere in Eagle’s Minimum Essential Medium (EMEM) containing L-glutamine (Gibco, Life Technologies, Carlsbad, CA, USA) with the addition of 10% fetal bovine serum (FBS) decomplemented in the bath at 56 °C for 20 min (Lonza Group Ltd., Switzerland) and 1% solution of penicillin and streptomycin (Gibco, Life Technologies Italy, Monza (MB), Italy).

U373-MG (Uppsala) cells were purchased from ECACC and maintained in EMEM (EBSS) medium (EuroClone, Pero (MI), Italy), 2 mM glutamine, 1% nonessential amino acids (NEAAs), 1 mM sodium pyruvate (NaP) and 10% fetal bovine serum (FBS) (Gibco, Life Technologies Italy, Monza (MB), Italy). Cells were infected with lentiviral luciferase vector pRRLSIN.cPPT.RFPL4b.Luciferase (Addgene, Watertown (MA), USA), which allows stable expression of luciferase for in vivo tumor growth monitoring.

### 3.2. Methods

#### 3.2.1. Preparation of Freeze-Dried SANPs

PEGylated cationic liposomes (PL) (2.5 mg/mL) (DOTAP/chol/DSPE-PEG_2000_ 1:1:0.5 weight ratio) were prepared by hydration of a thin lipid film, followed by extrusion. 1 mL of PLs was mixed with 5 mg/mL of Tf dissolved in buffer solution at pH 8, at room temperature for 15 min, thus obtaining a colloidal dispersion here named PL-Tf. Then, calcium phosphate nanoparticles (CaP NPs) were prepared and complexed with zoledronic acid (ZOL). Briefly, an aqueous solution of dibasic hydrogen phosphate (10.8 mM) was added dropwise to an aqueous solution of calcium chloride (18 mM) under continuous magnetic stirring. After 10 min of reaction, the resulting dispersion was filtered twice through a 0.22 μm filter (Sartorius). CaP encapsulating ZOL (CaPZ) were prepared by mixing 500 µL of CaP with 91 µL (0.4 mg) of an aqueous ZOL solution (0.125 M) in phosphate buffer at pH 9.5 for 15 min. Finally, SANPs were obtained by mixing the PL-Tf complex with CaPZ at room temperature for 15 min. The final product was then filtered through a 0.22 μm filter. SANPs were prepared in the presence and in the absence of cryoprotectants. Thus, in the case of freeze-dried SANPs, mannitol, trehalose or sucrose were added at 5% w/w with respect to the theoretical solid component of SANPs.

#### 3.2.2. Differential Scanning Calorimetric Analysis (DSC)

Thermoanalytical tests were carried out using a TA Q20 differential scanning calorimeter (TA Instruments, USA) on cryoprotectant solutions (5% *w*/*w*). Experiments were carried out on sucrose, trehalose and mannitol solutions (5% *w*/*w* with respect to the theoretical solid mass of SANPs). For each experiment, 15 μL of each solution was placed in a hermetically sealed DSC pan. The scans were performed under an inert nitrogen atmosphere (flow rate: 50 mL/min) using the following steps: (i) samples were equilibrated at 40 °C and cooled to −60 °C at 2 °C/min; (ii) an isotherm for 10 min was imposed; (iii) samples were heated to 60 °C. Heating and cooling rates were 2 °C/min. Onset and peak temperatures, along with variations in enthalpy (∆H), were observed. Deionized water was used as a control. DSC traces were taken on at least three replicas.

#### 3.2.3. Lyophilization

Before lyophilization, the samples were filtered on 0.2 µm acetate cellulose filters (Sartorius) and frozen using liquid nitrogen to ensure the annealing of amorphous water prior to lyophilization. Subsequently, aliquots (1.7 mL) of the product were poured into separate vials. The lyophilization process was carried out at a pressure of 0.13–0.2 mbar and with a temperature between −80 and −50 °C, for 24 h (Lyovapor L-200 Pro, Büchi Italia s.r.l).

#### 3.2.4. Particle Size Determination and Superficial Charge

The mean diameter, polydispersity index (PI) and zeta potential (ζ) of SANPs before and after freeze-drying and reconstitution with water were determined using a Zetasizer Ultra (Malvern Panalytical, Malvern, UK). Results were averaged over three measurements from independent batches.

#### 3.2.5. Cell Proliferation Assay

MTT assays were performed to assess cell viability. In detail, cells were plated at the density of 2.0 × 10^3^ cells/well in 96-well microtiter plates. After 24 h, cells were exposed to various concentrations of different formulations of SANPs to test their cytotoxicity. After 72 h, 10 μL of MTT (1 mg·mL^−1^) in 200 μL of medium was added to the cells in each well. After 3 h incubation at 37 °C, the medium was withdrawn, and then the formazan crystals were solubilized by adding 100 μL of DMSO and by mixing in an orbital shaker for 20 min. Absorbance at 570 nm was measured using a plate reader. Experiments were performed in triplicate. Data were expressed as mean value ± the standard deviation calculated for at least three repeats.

#### 3.2.6. In Vivo Study

Male nude (nu/nu) mice, 6 weeks old and weighing 24–26 g, were purchased from Envigo S. Pietro al Natisone (Udine). All animal experimentation procedures were approved by the Italian Ministry of Health (authorization No. 386/2020-PR, released date 30/04/2020) and followed national and international directives (D.L. March 4, 2014, No. 26; directive 2010/63/EU of the European Parliament and European Council; Guide for the Care and Use of Laboratory Animals, United States National Research Council, 2011).

Mice were anesthetized and injected intracranially with 2.5 × 10^5^ U373-MG UPPSALA LUC glioblastoma cells/mouse, through the center-middle area of the frontal bone to a 2-mm depth, using a 0.1 mL glass microsyringe and a 27-gauge disposable needle [18]. After intracranial implantation, the drinking water was supplemented with Metacam (meloxicam) to control postoperative pain and inflammation. After 7 days, mice were imaged using the IVIS imaging system 200 series (Caliper Life Sciences, Hopkinton, MA, USA). Briefly, mice were anesthetized with a combination of tiletamine–zolazepam (Telazol, Virbac, Carros, France) and xylazine (xylazine/Rompun BAYER) given intramuscularly at 2 mg/kg. Then mice were injected intraperitoneally with 150 mg/kg D-luciferin (PerkinElmer) and imaged in the supine position 10 min after luciferin injection. On day 8, mice were randomized and divided into three groups (untreated, SANPs and lyophilized SANPs) and the treatment was started. Mice were treated intravenously (i.v.) at 20 μg ZOL/mouse/day with SANPs three times a week for 3 consecutive weeks.

Imaging was performed at baseline (day of tumor cell injection) and several times during the experiment. Data were acquired and analyzed using the Living Image software version 4.3 (PerkinElmer Italy S.p.A., Milan, Italy).

Tumor growth was monitored by photon analysis. Complete tumor regression was defined as an undetectable bioluminescent signal in the brain of mice, lasting for at least four weeks. Animals were closely monitored by visual inspection and weighed daily from the start of treatment and sacrificed when evident signs of tumor burden appeared (especially weight loss >20% and severe neurological dysfunction).

## 4. Conclusions

This study explored an additional evolution of the SANP technology, with the aim of reducing the complexity of the preparation protocol and at the same time increasing the stability of the starting components. In particular, the purpose of the study was to provide a ready-for-use formulation starting from a freeze-dried powder to be reconstituted by the addition of the solvent immediately before administration. DSC studies, performed on different cryoprotectants, indicated that sucrose is the most suitable excipient and provides satisfactory protection of the formulation during lyophilization. This study also shed light on the pivotal freeze-drying setup parameters. Newly designed SANPs showed a high ability to deliver ZOL in GBM cells, as suggested by the significant inhibition of cell growth even at very low concentrations. In addition, the outcomes of experiments performed in an orthotopic model of GBM clearly indicate that the newly developed SANP formulation maintained its ability to induce tumor growth inhibition, stabilization of the disease and, more importantly, tumor regression in 1/6 mice. Taken together, results showed that the modified SANP formulation displayed a promising therapeutic efficacy, paving the way for additional studies devoted to the optimization of the production process from an industrial standpoint.

## Figures and Tables

**Table 1:** Thermal parameters of formulations containing mannitol, trehalose and sucrose.

Thermal Parameters of SANP Formulations in the Presence of Cryoprotectants					
Phase	Parameter	Water	Mannitol	Sucrose	Trehalose
**Heating**	Left limit (°C) ± SD *	-	−27.8 ± 1.6 *	-	-
	Peak (°C) ± SD *	-	−20.4 ± 2.2 *	-	-
	Right limit (°C) ± SD *	-	−14.3 ± 2.0 *	-	-
	Onset (°C) ± SD *	-	−26.2 ± 2.1 *	-	-
	∆H (J/g) ± SD *	-	3.21 ± 0.38 *	-	-
**Heating**	Left limit (°C) ± SD *	−0.23 ± 0.10 *	−9.24 ± 1.14 *	−9.67 ± 0.6 *	−10.0 ± 0.4 *
	Peak (°C) ± SD *	2.58 ± 0.01 *	1.99 ± 0.38 *	1.35 ± 0.30 *	1.73 ± 0.22 *
	Right limit (°C) ± SD *	8.26 ± 0.83 *	7.52 ± 0.40 *	7.57 ± 1.10 *	8.14 ± 1.72 *
	Onset (°C) ± SD *	−0.57 ± 1.55 *	−1.60 ± 0.10 *	−1.65 ± 0.09 *	−1.24 ± 0.03 *
	∆H (J/g) ± SD *	338 ± 13 *	297 ± 13 *	283 ± 9 *	296 ± 17 *

* Standard deviations were calculated for *n* ≥ 2 independent batches; water was used as a reference.

**Table 2:** Mean diameter, polydispersity index and zeta potential of SANP formulations without or with different kinds of cryoprotectants.

Technological Features of SANPs before and after Lyophilization				
Cryoprotectant	Lyophilization	Mean (nm) ± SD *	PI ± SD *	PZ (mV) ± SD *
**-**	Before	131.0 ± 10.7 *	0.143 ± 0.020 *	+5.9 ± 0.7 *
	After	244.2 ± 14.0 *	0.466 ± 0.030 *	+6.4 ± 0.9 *
**Sucrose**	Before	162.4 ± 6.8 *	0.216 ± 0.020 *	+8.1 ± 2.0 *
	After	159.6 ± 15.4 *	0.245 ± 0.030 *	+7.4 ± 0.7 *
**Trehalose**	Before	123.3 ± 4.2 *	0.149 ± 0.010 *	+5.4 ± 0.6 *
	After	380.0 ± 7.7 *	0.514 ± 0.300 *	+6.5 ± 1.5 *

* Standard deviations were calculated for at least three independent batches.

**Table 3:** Mean diameter, polydispersity index and zeta potential of SANPs 1 and SANPs 2 formulations before and after lyophilization.

Characteristics of SANPs Lyophilized in the Presence of Sucrose				
Formulation	Lyophilization	Mean (nm) ± SD *	PI ± SD *	PZ (mV) ± SD *
**SANPs 1**	Before	134.4 ± 3.3 *	0.126 ± 5.3 *	+5.3 ± 0.1 *
	After	145.6 ± 15.4 *	0.245 ± 0.03 *	+8.9 ± 3.3 *
**SANPs 2**	Before	110.8 ± 6.5 *	0.165 ± 0.040 *	+8.2 ± 3.2 *
	After	91.3 ± 6.3 *	0.220 ± 0.030 *	+12.0 ± 2.3 *

* Standard deviations were calculated for at least three independent batches.

**Table 4:** Antitumor efficacy of SANP formulations against orthotopic GBM.

In Vivo Antitumoral Effect of SANPs			
Treatment Groups *	Tumor Volume Inhibition ^&^	Stable Disease ^§^	Complete Response ^$^
**SANPs 1**	58	3/6	1/6
**SANPs 2-L**	50	3/6	1/6

* Following U373-MG (Uppsala) LUC orthotopic injection, mice were treated with SANPs 1 or SANPs 2 iv at 20 μg/mouse/day for three days a week for three total weeks. ^&^ Tumor volume inhibition was calculated as the nadir of the effect (day 30) by the following formula: (photons treated mice/photons untreated mice -1) × 100. Standard deviation (SD) was calculated on day 30 referring to the numbers of photons. (SDs of SANPs 1 and SANP 2-L were 7.8 × 10^4^ and 9.7 × 10^4^, respectively). ^§^ Stable disease was the observation of the same level of bioluminescent signal for two weeks. ^$^ Complete response was defined as mice with observed disappearance of bioluminescent signal for at least four weeks after treatment start.

## Data Availability

The data presented in this study are available on request from the corresponding author.
